# Noninvasive Technique to Monitor the Pressure under a Cast: A Mobile Application-Friendly Bluetooth Pressure Sensor

**DOI:** 10.1155/2022/9093612

**Published:** 2022-10-31

**Authors:** Mahir Mahirogullari, Serkan Surucu, Mehmet Halis Cerci, Mahmud Aydin, Abdulkadir Kayikli, Oguzhan Gunduz

**Affiliations:** ^1^Şişli Memorial Hospital, Piyalepaşa Blv. 34385, Şişli, İstanbul, Turkey; ^2^Yale University, Department of Orthopaedic and Rehabilitation, New Haven 06510, CT, USA; ^3^Haseki Education Research Hospital, Uğur Mumcu Mahallesi, Belediye Sokak, No: 7, Sultangazi, İstanbul, Turkey; ^4^Hayriya Bilişim ve Sağlık Teknolojileri AŞ, Teknopark, Pendik, İstanbul 34976, Turkey; ^5^Center for Nanotechnology&Biomaterials Application and Research (NBUAM), Marmara University, Kadikoy, İstanbul, Turkey

## Abstract

**Aim:**

The purpose of this study was to design a sensor that could monitor the skin-cast contact surface pressure (SCCSP) of a limb under a cast and inform the user via a mobile application when the pressure increases.

**Methods:**

In this experimental study, an infant sphygmomanometer cuff was initially placed on the forearm of 10 volunteers. A pressure sensor with a Bluetooth chip was then placed on the volar aspect of the forearm. Short arm plaster was applied with synthetic cast material. The SCCSP under the plaster was measured by the sensor and the measured values were transmitted to a mobile application via a Bluetooth chip. The mobile application processed the data from the chip and converted it to mmHg.

**Results:**

Intracompartmental pressure (ICP) values were categorized as 0, 10, 20, 30, 40, 50, 60, and 75 mmHg. The highest SCCSP was 75 mmHg CP, while the lowest was 0 mmHg CP. The correlation coefficient of the mean pressure values was 0.993 (*p* ≤ 0.001) (SD 0.002, range 0.989–0.997), and there was a significant relationship between ICP and SCCSP values (*p* ≤ 0.05).

**Conclusion:**

We can monitor SCCSP, detect limb swelling, and notify the user via a mobile application by using Bluetooth pressure sensors.

## 1. Introduction

Various methods of fracture treatment have been attempted throughout history; nevertheless, closed reduction and casting are the most often utilized procedures [1, 2]. Closed reduction of the fracture promotes healing in a timely and appropriate manner and minimizes complications [3]. Following the fracture reduction, it is critical to mold the cast to the extremity to prevent excessive movement of the fractured bone and to maintain fracture reduction [4]. Increased swelling of the extremity following trauma, increased molding of the cast and tight casting following reduction can all result in circulation complications, nerve entrapment, skin necrosis, and possibly compartment syndrome under the cast [5, 6].

Although clinical findings have an important role in the diagnosis of compartment syndrome, many authors have described invasive and noninvasive intracompartmental pressure (ICP) monitoring for the diagnosis of compartment syndrome [7, 8]. Since ICP monitoring is an invasive method, it cannot be performed when the patient's limb is in a cast. Therefore, noninvasive techniques have been developed to measure the skin surface pressure of the limb in a cast [9, 10].

The purpose of this study was to design a sensor capable of monitoring the skin-cast contact surface pressure (SCCSP) of a limb under a cast and notifying the user via a mobile application in the case of swelling that could impair limb circulation. We hypothesized that the pressure under the cast was correlated to the pressure in the cuff, namely, swelling.

## 2. Methods

### 2.1. Cast Application and Pressure Measurement

**Figure 1:**
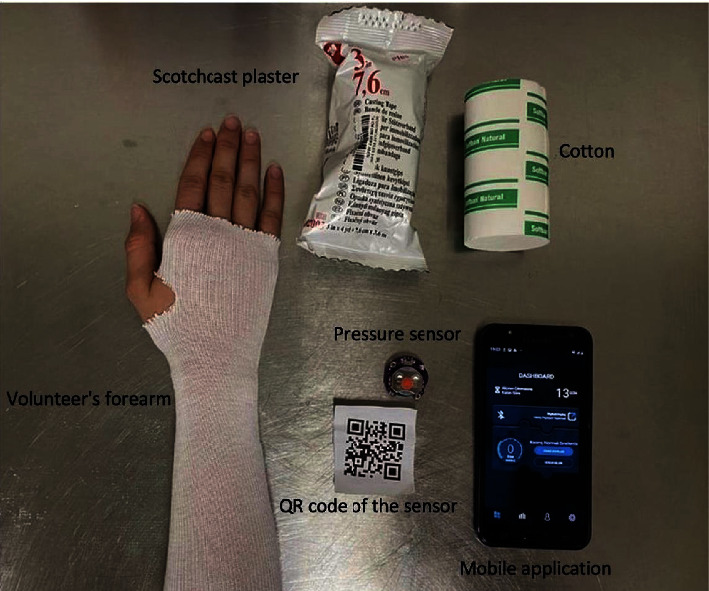
Materials required for skin-cast contact surface pressure measurement.

This study was approved by the local institutional review board and conducted in accordance with the principles of the Declaration of Helsinki (IRB No: 2021-314). In this experimental study, after providing detailed information about the procedure and obtaining written consent from all individuals, a baby sphygmomanometer cuff (Cuff-mounted sphygmomanometer HS-20C, Honsun, China) was first placed on the forearm of 10 volunteers. The stockinet (BSN Medical, BSD City, Indonesia) was then dressed. A pressure sensor (Force Sensing Resistors®, Nanjing, China) with a Bluetooth chip (SimpleLink™ Bluetooth®, Texas, USA) was placed on the volar aspects of the forearm. The forearm and pressure sensor were wrapped with synthetic cast padding (3M Health Care, St. Paul, Minnesota, USA) with 50% overlap at each pass (resulting in two layers of cotton), followed by Scotchcast plaster (Scotchcast™, 3M Health Care, St. Paul, Minnesota, USA) with 50% overlap at each pass (ultimately four layers of plaster), and the plaster was left to harden for 30 min (Figure 1). The SCCSP under the cast was measured by the sensor, and the measured values were transmitted to a mobile application via a Bluetooth chip. The mobile application processed data from the chip and converted it into mmHg. First, the baseline SCCSP was determined after casting. Then, the cuff was inflated gradually to increase the pressure, and the SCCSP displayed by the mobile application was noted. Data from the participants were pooled and classified as 0, 10, 20, 30, 40, 50, 60, and 75 mmHg according to the cuff pressure.

### 2.2. Bluetooth Pressure Sensor and Mobile Application

The Bluetooth pressure sensor used in this study measured 25 mm in diameter and 15 mm in thickness. It works on a GP® CR2032 battery, which has a lifespan of 2–5 years. It has a CC2541 Bluetooth chip and a force sensor attached to the legs of the chip. When pressure is applied to the sensor, the chip converts it into an electric signal and sends it to a mobile application (Safecast™) via Bluetooth.

**Figure 2:**
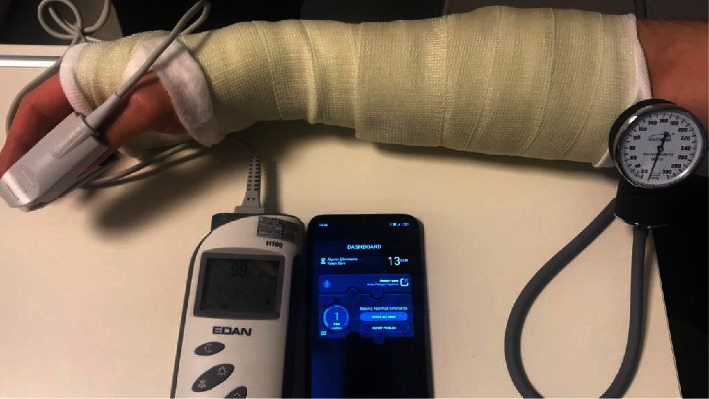
Determination of cut-off values with pulse oximetry.

**Figure 3:**
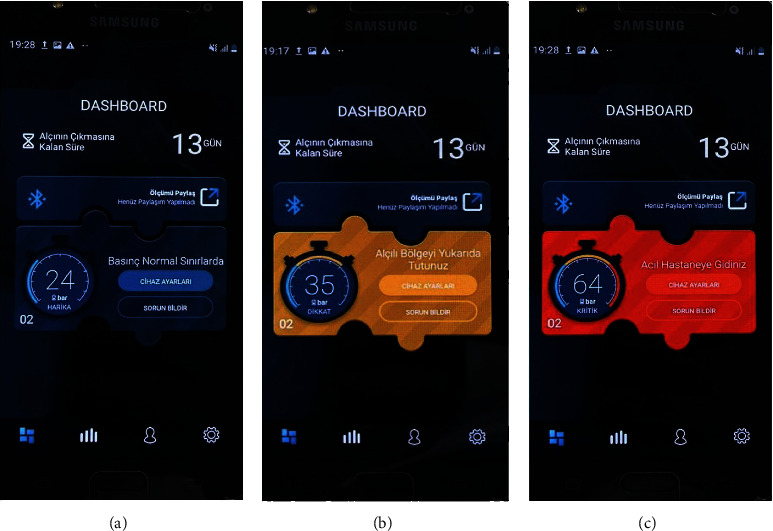
Three categories of skin-cast contact surface pressure levels in the application. (a) The first category is the “normal pressure category” with pressure values of 0–50 mmHg and is shown in “green.” (b) The second category is defined as pressure values of 50–75 mmHg, indicating increased pressure and a moderate risk of developing compartment syndrome, and is indicated by “yellow.” (c) The third category is defined as pressure values > 75 mmHg, indicating increased pressure and a high risk of developing compartment syndrome, and is indicated by “red.”

The mobile app instantly displays the pressure applied to the sensor. Cut-off values were determined by evaluating capillary refill time and circulation in the distal extremity by pulse oximetry (Figure 2). According to these values, three pressure categories were created for this application. The first category is the “normal pressure category” with pressure values of 0–50 mmHg and is shown in “green” (Figure 3(a)). The second category was defined according to the 50 mmHg pressure value at which oxygen saturation began to decrease in pulse oximetry. It is defined as pressure values of 50–75 mmHg, indicating increased pressure and the risk of developing complications, and is indicated by “yellow.” When this category of pressure is detected by the sensor, the app sends a notification to the user: “Please do not forget to lift your limb!” (Figure 3(b)). The third category was defined according to the pressure value of 75 mmHg, in which the capillary refill time increased for more than 3 seconds. It is defined as pressure values >75 mmHg, indicating increased pressure and a high risk of developing circulatory disorders, and is indicated by “red.” When the pressure in this category is sensed by the sensor, the app prompts the user with a “Please, Go to the Nearest Emergency Room!” sends alert (Figure 3(c)).

### 2.3. Statistical Analysis

The normality of the data was examined using the Kolmogorov–Smirnov test, and it was determined that the data had a normal distribution (*p* > 0.05). One-way analysis of variance was performed in repeated measurements of the data and using Mauchly's sphericity test, it was determined that the variances were not equal (*p* < 0.05). Therefore, pressure comparisons were made using the Greenhouse–Geisser test because *Ɛ* was < 0.75 in the comparison of pressures. Additionally, Spearman's correlation analysis and regression analysis were performed for data analysis. All statistical analyses were performed using SPSS software (Version 20.0; IBM Corp., Armonk, NY, USA).

## 3. Results

**Figure 4:**
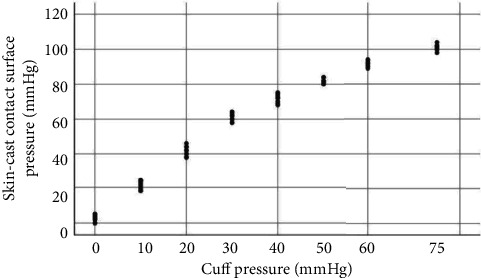
Graph showing the range of skin-cast contact surface pressure values (mmHg) relative to the cuff pressure values (mmHg).

Data from participants were collected and classified as 0, 10, 20, 30, 40, 50, 60, and 75 mmHg based on CP. The highest SCCSP was detected at 75 mmHg CP, whereas the lowest SCCSP was detected at 0 mmHg CP. There was a significant difference in the SCCSP measured at varied cuff pressure measurements (*F* = 7495.988; *p* ≤ 0.001) (Table 1). The range of SCCSP values (mmHg) based on cuff pressure values is demonstrated in Figure 4.

The correlation between participants' ICP and SCCSP mean values was determined using Spearman's correlation analysis. The results of the analysis are presented in Table 2. According to the analysis, the correlation coefficient of the mean pressure values was 0.993 (*p* ≤ 0.001) (SD 0.002, range 0.989–0.997), and there was a significant relationship between CP and SCCSP values (*p* ≤ 0.05).

The regression analysis to determine the relationship model between SCCSP measurements and ICP pressure levels was found to be statistically significant (*F* = 1574.51; *p* ≤ 0.001; *p* ≤ 0.01) (Table 3). The correlation between ICP and SCCSP measurements is quite good, and it is seen that ICP's explanation rate of SCCSP is 95.3%.

A statistically significant difference was found between SpO_2_ measurements according to pressure levels (*p* < 0.01). When the significances are examined; SPO_2_ measurements taken at ICP baseline, 30 and 50 mmHg pressure levels were found to be significantly higher than the measurements at 60 mmHg pressure (*p* ≤ 0.001; *p*=0.004; *p*=0.008; *p* ≤ 0.01). SpO_2_ measurements at ICP baseline, 30 and 50 levels do not differ significantly (Table 4).

## 4. Discussion

Acute compartment syndrome is a limb-threatening emergency that occurs most often after fracture [11] and cast application [12, 13]. Clinical findings such as severe pain, weak pulse, reduced mobility, numbness, or pallor of the affected limb have low sensitivity (13%–64%) compared with ICP monitoring (94%) for diagnosing acute compartment syndrome, and they can be absent in some cases [14, 15].

While early diagnosis of acute compartment syndrome is critical, invasive ICP monitoring is extremely difficult. Therefore, we utilized a Bluetooth pressure sensor to monitor SCCSP as an indirect predictor for ICP and a mobile application to notify the patient of increased swelling, thereby preventing a delayed diagnosis of compartment syndrome. The purpose of this study was not to measure compartment syndrome but to detect swelling, one of the early symptoms, during the initial stages. This study differs from others in its field because it was conducted in healthy volunteers, i.e., in vivo, and it utilizes a newly designed pressure sensor to make noninvasive measurements. In this preliminary study, capillary refill time and circulation in the distal extremities were evaluated using pulse oximetry. In this application, three pressure categories were defined based on these values.

In the experimental study conducted by Uslu and Apan, skin surface pressures and compartment mental pressures were measured under plaster in the forearm model and the posterior tibial compartment syndrome model of rabbits. The results revealed that the correlation between compartment pressure and skin surface pressure was high/excellent, implying that skin surface pressure can be used to monitor compartment pressure under a plaster cast [10]. Yuri et al. [16] developed a new method for noninvasively monitoring intracompartmental pressure using shear wave elastography for a pressure increase in the lower extremity in healthy volunteers. In another study, Chang et al. [17] designed a portable and convenient noninvasive pressure monitoring system for compartment detection and demonstrated that compartment pressure can be measured in vitro with finite element analysis. In this study, we determined the amount of swelling in the patient's forearm under the plaster by inflating the cuff; therefore, we examined the relationship between cuff pressure and SCCSP and discovered a high correlation. Since previous studies have revealed that forearm compartment syndrome is more common in the forearm, we placed the sensor only on the volar side of the forearm [18, 19].

Sawyer et al. investigated the reasons for emergency department (ER) visits for cast-related issues. They discovered that each visit necessitated straightforward procedures that might have been handled within clinic hours. This showed that patient education, triage, and follow-up in fracture clinics could prevent unnecessary ER visits and decrease the financial burden on the healthcare system [20]. In this study, we used a Bluetooth pressure sensor to monitor the SCCSP. This pressure sensor, which shows the SCCSP through a mobile application, could reassure the patient about normal pressure when there is no risk and prevent unnecessary ER visits. A new version of this method that allows for more comprehensive follow-up, not only by patients but also by physicians and healthcare facilities, may be developed.

One of the limitations of the study was that it was an experimental study. Additional clinical research is needed to assess the validity of pressure measurements, false-positive and false-negative results, and measurement cut-off values. This can help to ensure that false-negative signals do not lead to an unwarranted sense of trust. This was a preliminary study, and we think that the sensor will be acceptable for orthopedic treatments following successful clinical testing. Another study limitation was the sensor's placement on the volar side of the forearm. We intend to place sensors in additional locations in future clinical research, considering other compartments.

## 5. Conclusion

We designed a Bluetooth pressure sensor kit that can measure SCCSP under a cast, monitor limb swelling, and notify the user with a mobile application. Additionally, we believe that this noninvasive method will help reduce the frequency of hospital admissions.

## Figures and Tables

**Table 1:** Mean SCCSP with respect to the mean CP.

Mean CP (mmHg)	Mean SCCSP (mmHg)	SD	CI (95%)
0	2.80	0.57	1.5–4.1
10	21.30	0.78	19.5–23.1
20	41.20	0.90	39.1–43.2
30	61.30	0.70	59.7–62.9
40	72.00	0.76	70.3–73.7
50	82.00	0.49	80.9–83.1
60	91.70	0.54	90.5–92.9
75	101.10	0.53	99.9–102.2

CI: confidence Interval, CP: cuff pressure; SCCSP: skin-cast contact surface pressure; SD: standard deviation.

**Table 2:** Spearman's correlation analysis between CP and SCCSP.

			CP	SCCSP
Spearman's rho	CP	Correlation coefficient	1.000	0.993^*∗∗*^
		Sig. (two-tailed)	—	0.000
		N	80	80
	SCCSP	Correlation coefficient	0.993^*∗∗*^	1.000
		Sig. (two-tailed)	0.000	—
		N	80	80

^*∗∗*^Correlation is significant at the 0.01 level (two-tailed). CP, cuff pressure; SCCSP, skin-cast contact surface pressure.

**Table 3:** Regression model of the effect of CP pressure levels on SCCSP measurements.

Dependent variable	Independent variable	*ß*	*t*	*p*	*F*	Model (*p*)	*R * ^2^
SCCSP	Constant	11.832	8.235	≤ 0.001	1574.51	≤ 0.001	0.953
	ICP	1.329	39.680	≤ 0.001			

**Table 4:** Variation of SpO_2_ measurements in ICP pressure levels.

CP (mmHg)	Median SPO_2_ (mmHg)	IQR
		Q1–Q3
Base	97.0	96.0–98.0
30	97.0	95.8–97.5
50	97.0	95.8–97.3
60	92.0	91,5–92.3
*p* (Friedman test)	0.001	
Post hoc dunn bonferroni test	CP 60< CP 0, 30, 50	

IQR: interquartil range Q1: %25 percentil q3: %75 percentil.

## Data Availability

The data used to support the findings of this study are available from the corresponding author upon request.

## Additional Points

*What Is Known about This Study* (i) Increased swelling of the extremity after trauma, increased molding in the cast, and tight casting following reduction can cause circulatory complications and possibly compartment syndrome under the cast. (ii) Although clinical findings play an important role in the diagnosis of compartment syndrome, many authors have defined invasive and noninvasive intracompartmental pressure monitoring for the diagnosis of compartment syndrome. *What Is New about This Study*. (i) In this study, we designed a sensor that can monitor the skin-cast contact surface pressure (SCCSP) of a limb under plaster and inform the user via mobile application when the pressure increases.
